# 70274 TL1 team approach to investigating the adhesin gene fimH in adherent invasive E. coli induced inflammation and colorectal cancer development

**DOI:** 10.1017/cts.2021.675

**Published:** 2021-03-30

**Authors:** Rachel C Newsome, Qin Yu, Yoshitaka Murota, Derek Hood, Duy Nguyen, Ryan A Smolchek, Juan M Uruena, W Gregory Sawyer, Christian Jobin

**Affiliations:** 1 University of Florida Department of Medicine; 2 University of Florida Department of Mechanical and Aerospace Engineering

## Abstract

ABSTRACT IMPACT: We are developing the 3D perfusion system for use with patient-derived bacteria to further characterize the mechanism behind bacterial-induced inflammation and cancer. OBJECTIVES/GOALS: We previously reported the adherent invasive E. coli NC101 promote colorectal cancer (CRC) in mice. FimH, a mannose-specific adhesin on type 1 fimbriae, is involved in bacterial surface adhesion. Herein, we investigated the role of FimH in E. coli NC101-induced adherence and carcinogenesis in a novel 3D perfusion culture imaging plate. METHODS/STUDY POPULATION: E. coli NC101 gene fimH was deleted byï ŲRed Recombinase System. Biofilm formation was assessed by crystal violet and congo red staining. 5 dpf (wild-type strain) zebrafish embryos were infected in 6x107 cfu/ml wild type (WT) or fimH-deleted (ï ,,fimH) E. coli NC101 for 24hr and gut dissected for bacterial culture. A 2D/3D infection culture system for IEC-6 and HT-29 cells was infected for 4 hr and imaged and then DNA damage examined by comet assay, cell cycle andÎ^3^H2AX accumulation. Germ-free (GF) Il10-/- (colitis) mice were orally gavaged with 108 cfu WT orï ,,fimH E. coli NC101 for 16 weeks. E. coli colonization were quantified by plate culture and qPCR. Lipocalin2 was quantified by ELISA. PCNA and β-catenin were evaluated by immunohistochemistry (IHC). RESULTS/ANTICIPATED RESULTS: Biofilm formation was reduced by more than 40% (p<0.05) in E. coli NC101ï ,,fimH compared to WT strain. Zebrafish larvae showed a 41% decrease in intestinal colonization ofï ,,fimH compared to WT (p<0.05). E. coli NC101-induced DNA damage was reduced by 67% (p<0.0001) in HT-29 cells infected withï ,,fimH compared to WT strain. Using the 3D infection system, a 46% decrease in yH2AX (p<0.05) and 42% decrease in G2 cell cycle arrest (p<0.05) was observed inï ,,fimH infected IEC-6 cells compared to WT strain. Furthermore, ï ,,fimH infected Il10-/- mice showed decreased colonization (p<0.01), decreased intestinal inflammation (p<0.05), decreased stool lipocalin2 level (p<0.01), and reduction of PCNA positive cells in the intestine (p<0.05) compared to mice infected with WT strain. DISCUSSION/SIGNIFICANCE OF FINDINGS: Adhesin protein FimH is required by E. coli NC101 to colonize and promote colitis and carcinogenesis both in a 3D perfusion culture and in mice and may serve as potential therapeutic target.

